# Bio-surface modification of polyester containing fabrics using lipase for post-multifunctionalization

**DOI:** 10.1038/s41598-025-20236-x

**Published:** 2025-10-14

**Authors:** Nabil A. Ibrahim, Hala A. Amin, Hanan M. Ahmed, Mohamed S. Abdel-Aziz, Ahmed A. Hamed, Mohamed A. Yassin, Basma M. Eid

**Affiliations:** 1 Institute of Textile Research and Technology, National Research Centre, 33 EL Buhouth St, P.O. 12622, Dokki, Giza Egypt; 2 Chemistry of Natural and Microbial Products Department, National Research Centre, Dokki, Giza, Egypt; 3 Microbial Chemistry Department, National Research Centre, P.O. 12622, Dokki, Giza Egypt; 4 Chemical Industries Research Institute, National Research Centre, El Buhouth St. 33, Giza, 12622 Egypt; 5 Leibniz-Institut für Polymerforschung Dresden e.V, Hohe Straße 6, 01069 Dresden, Germany

**Keywords:** Fungal lipase, Preparation and characterization, Potential textile application, Surface modifications, Post-functionalization, Green multifunctionalized textiles, Oil stains removal, Biotechnology, Materials science

## Abstract

An eco-friendly, sustainable approach for pre-surface modification of various textile substrates using locally produced fungal lipase from *Aspergillus niger* HANAN-EGY strain, followed by post-functional finishing using vanillin and/or zinc oxide nanoparticles (ZnONPs) as green functional additives, citric acid/sodium hypophosphite (CA, SHP) as ester-crosslinking agent, and the pad-dry-microwave fixation technique was developed. The imparted antibacterial, anti-UV, and aroma fragrance release functional properties, along with the change in %N of wool containing fabrics, loss in weight, as well as the surface roughness, were evaluated. SEM and EDX analyses were also performed on selected finished fabric samples. The data so obtained demonstrated that the extent of pre-surface modification and subsequent multifunctionalization is governed by the lipase dose, type of substrate, as well as kind and concentration of functional additive. The adoption and implementation of the suggested environmentally sound strategy results in the production of green, sustainable, antimicrobial, anti-UV, and fragrance-releasing textiles. On the other hand, the produced fungal lipase could be used to remove oil stains.

## Introduction

The 2030 Agenda for Sustainable Development and rising consumer demands drive environmental sustainability in the textile sector^1^. Balancing high productivity with reduced environmental impact is crucial, and cleaner production offers a viable approach by improving traditional manufacturing processes^1,2^.

Recently, much interest has been given to the adoption and implementation of environmentally sound technologies like bio- and nano- technologies in textile wet-processing to develop an eco-friendly, sustainable processes and to produce high quality, high added-value durable and multifunctional textile products taking in consideration both the ever-growing environmental concerns and the ever increasing textile consumer needs^3–6^. Many R&D efforts were done to achieve the aforementioned goals^7–9^. However, more efforts are still required.

Enzyme technology is increasingly used in the textile industry for wet processes, aftercare, and pollution management, promoting eco-friendliness by reducing waste and enhancing treatment methods^10^. This green approach balances resource use with economic growth, enabling the creation of innovative, eco-friendly textile products at lower costs. Selecting appropriate biocatalysts, enzymes, and fibers, along with optimal treatment conditions, is essential for producing high-quality textiles while safeguarding the environment^3,11^. Enzymes such as amylase, laccase, cellulase, pectinase, catalase, protease, and lipase are extensively used in textile manufacturing^12–16^.

Nanotechnology is gaining traction for its economic potential, particularly in the textile industry, which is adopting nanoparticles to meet consumer demand for durable and sustainable clothing^17^. Zinc oxide nanoparticles (ZnONPs) are safe and stable at high temperatures and during photocatalytic oxidation, making them attractive for applications in sunscreens, UV absorbers, and more^18,19^. They are also cost-effective for biomedical uses^17^. Efforts to create ZnONPs with unique structures have led to its use in textiles for multifunctional properties like antibacterial, self-cleaning, flame-retardant, and UV protection, addressing environmental concerns and consumer needs^20^. Applying ZnONPs to cotton textiles presents a promising strategy for creating a microbe-free environment, owing to their non-toxic and self-cleaning characteristics^21^.

Lipases, classified as triacylglycerol acyl hydrolases (EC 3.1.1.3), facilitate the hydrolytic breakdown of triacylglycerols into fatty acids and glycerol^22^. Lipases of microbial origin are more cost-effective than those of animal or plant origin and thus are broadly utilized in the detergent and textile industries^23^. Additionally, lipases are primarily utilized in the textile sector to degrease raw textile materials and enhance their performance^24^. Lipases play a crucial role in the removal of size lubricants, improving the absorbency of fabrics and facilitating a more uniform dyeing process. The lipase derived from *Aspergillus oryzae* is particularly effective in modifying polyethylene terephthalate (PET) fabrics, as it increases their polarity and anti-static properties, thereby improving moisture retention and dye absorption^25^.

In continuous of our interest to develop an environmentally friendly multifunctional textile products using green materials and sustainable processing technique^7,8,26,27^, new approach was developed to fabricate multifunctional textile fabrics with remarkable performance and functionalities using the locally produced lipase enzyme, for pre-surface modification, followed by subsequent functional finishing using ZnONPs and/or vanillin as functional additives, citric acid/sodium hypophosphite, as an eco-friendly ester-crosslinking system, and the pad-microwave fixation technique was developed taking in consideration both the ever-growing consumer needs and the increasing ecological concerns.

## Materials and methods

### Microorganism and maintenance

The experimental culture used in lipase production was inoculated from a single slant (isolated from butter). The stock cultures of the experimental microorganism were maintained on a potato dextrose agar medium (PDA). All the slant cultures were stored in a refrigerator and regularly transferred every month. It was taxonomically identified as *Aspergillus niger* based on the shape of conidia and arrangement of spores on the mycelia (conidial ontogeny) according to Barnett and Hunter^28^. Further molecular identification of the fungal isolate will be performed using the 18 S rDNA technique.

## Fungal isolate molecular identification

Fungal isolate genomic DNA was extracted using Quick-DNA™ Fungal/Bacterial Microprep Kit (D6007, Zymo Research, Irvine, USA), which was then amplified using Maxima Hot Start PCR 2× Master Mix (K1051, Thermo Scientific, Bartlesville, USA) that contained all of the necessary reagents except primer and DNA template. The amplification process was carried out in PCR tubes containing 25 µL Master Mix, 5 µL template DNA, 1 µL (20 µM) of ITS1/ITS4 primers, and 18 µL nuclease-free water. The sequences of the ITS1 and ITS4 primers were 5ʻ-TCCGTAGGTGAACCTGCGG-3ʻ and 5-TCCTCCGCTTATTGATATGC-3, respectively. The following temperature profile was used for the PCR amplification: an initial step cycle for 10 min at 95 °C; 35 cycles each consisting of the following segments: denaturation at 95 °C for 0.5 min, primer annealing at 52 °C for 1 min, and extension at 72 °C for 1.5 min. Then, a final extension cycle at 72 °C for 10 min was carried out. PCR amplification products were purified using Gene JET™ PCR Purification Kit (K0701, Thermo Scientific, Bartlesville, USA). The DNA sequencing was carried out by GATC Biotech Company (Konstanz, Germany) with an ABI 3730xl DNA sequencer using forward and reverse primers. The resulting sequences were compared to the standard rDNA sequences in the GenBank database using the basic local alignment search tool (BLAST)^29^, available at the National Center for Biotechnology Information (NCBI) website (http://www.ncbi.nlm.nih.gov).

## Culture media

The growth medium used for inoculum preparation is composed of (%, w/v) dextrose, 1; malt, 0.3; yeast extract, 0.3; peptone, 0.5. The pH of the medium was initially adjusted to 7.0. For inoculum preparation, one slant was cultivated in a 250 mL Erlenmeyer flask containing 100 mL of growth medium and incubated for 3 days at 200 rpm and 30 °C.

For lipase production, five media were tested. Their composition is illustrated in Table 1.

**Table 1:** Composition of production media for lipase production.

Medium	Composition (%, w/v)	pH	Reference
M1	peptone, 1.0; olive oil (OO), 1; KH_2_PO_4_, 0.2; KCl, 0.05; MgSO_4_.7H_2_O, 0.05	6.0	^30^
M2	peptone, 1.5; yeast extract, 0.5; OO, 1; KH_2_PO_4_, 0.3; MgSO_4_.7H_2_O, 0.04	6.0	^31^
M3	peptone, 2; glucose, 0.5; OO, 1; MgSO_4_.7H_2_O, 0.075; KCl, 0.05; KH_2_PO_4_, 0.2	7.0	^32^
M4	wheat bran extract, 10; KH_2_PO_4_, 0.2; MgSO_4_.7H_2_O, 0.1; yeast extract, 4.5; OO, 2. Add 1 mL of trace solution, whose composition was FeSO_4_·7H_2_O (0.63 mg), MnSO_4_ (0.01 mg), ZnSO_4_ (0.62 mg), and distilled water up to a volume of 1 L	7.0	^33^
M5	sucrose, 0.5; OO, 2; NH_4_NO_3_, 0.12; KH_2_PO_4_, 0.1; MgSO_4_.7H_2_O, 0.005; CuSO_4_.5 H_2_O, 0.006; FeCl_3_, 0.1	7.0	^34^

The media, 100 mL of each production medium, were emulsified with 10% (w/v) Arabic gum in a homogenizer. The flasks were then inoculated with 5 mL of prepared inoculum and incubated on a reciprocal shaker (200 rpm) at 30 °C for 4 and 6 days. Fungal cells were separated from the broth by filtration through filter paper. The culture filtrate was collected for further lipase estimation.

## Optimization of the fermentation conditions

Five different media (M1-M5) were tested for lipase production by *A. niger* HANAN-EGY. Various nitrogen sources (yeast extract, corn steep liquor, soybean meal, baker’s yeast, (NH_4_)_2_SO4, and NH_4_Cl), and different oils (soybean oil, sunflower oil, waste frying oil, and jatropha oil by a one-factor-at-a-time method using M4 as the basal medium. Based on the results obtained from the one-factor-at-a-time method, suitable carbon (wheat bran, WB) and nitrogen sources (yeast extract, YE), as well as an enzyme inducer (OO), were employed for mathematical design. Three parameters, WB concentration, YE concentration, and OO concentration, were optimized using a central composite orthogonal design (CCO). A response surface CCO design was created for 3 continuous factors. The axial value can be selected, and the default for the orthogonal design is set at an axial value of 1.287. By having 2 center points and 1 replicate, an experimental design with 32 runs is established.

## Lipase assay

One mL of culture filtrate (after the separation of the microbial growth) was mixed with 2.5 mL of deionized water, 1 mL of 0.1 M Tris-HCl buffer (pH 7.5), and 3 mL of an emulsion of 10% (w/v) OO and 10% (w/v) Arabic gum in hot water^35^. The mixture was incubated for 2 h at 37 °C in a shaking water bath, after which 10 mL of 99% acetone was added to stop the reaction and promote the extraction of the free fatty acids. The resulting mixture was then titrated against 0.05 N NaOH using a thymolphthalein indicator. It should be noted that NaOH was previously standardized against standard 0.05 N HCl using the phenolphthalein indicator. Blanks obtained with frozen or boiled enzyme samples were subtracted and the activities were obtained in terms of U/ml of the enzyme solution. All determinations were carried out in triplicate. One unit of lipase activity was defined as the amount of enzyme that produces 1 µmole of free fatty acids per min under certain conditions.

### Pilot scale production

Lipase was produced on a pilot scale using a 7 L bioreactor (Bioflo-310 bioreactor, New-Brunswick Scientific Co., ltd, Germany). The bioreactor had a working volume of 4 L. It was equipped with a basal heating system, internal cooling coil, and exhaust condenser to maintain a temperature of 30 °C and prevent evaporation loss. Dissolved oxygen (DO) and pH probes measured dissolved oxygen and pH, respectively. The probes were calibrated before adding the media to the fermenter vessel, which was autoclaved in a vertical-type autoclave. A program was set to regulate the necessary parameters for the experiment design, ensuring that the bioreactor maintained the set values during the fermentation process^36^.

### Statistical analysis

All experiments were performed in triplicate, and data were expressed as mean ± standard deviation. Statistical optimization was designed and analyzed using JMP statistical software (Version 8. SAS Institute Inc., Cary, NC).

## Lipase pre-treatment of several substrates

Mill-scoured and bleached 100% polyester (160 g/m^2^), wool/PET (50/50, 165 g/m^2^), and cotton/PET (50/50, 240 g/m^2^) were bio-modified using the bio-prepared lipase enzyme (75 mL enzyme stock solution/150 mL water), at material to liquor ratio (MLR) 1/20 in IR-dyeing machine at 50 °C for 60 min After the enzymatic treatment, the bio-process was terminated by raising the temperature to 70 °C for 15 min, followed by thorough washing and drying.

### Functional finishing of the bio-modified fabrics

Portions of the bio-modified fabric samples were finished with ZnONPs (10 g/L), and vanillin (10 g/L) individually or combined, in the presence of citric acid (20 g/L), as a crosslinking agent, and sodium hypophosphite SHP (10 g/L), as a catalyst, at 30 °C for 20 min in a sonicator bath with 55 kHz frequency, followed by padding twice to 80% wet pick-up and microwave fixation at 400 W for 6 min.

#### Full evaluation of bio-modified and finished fabrics

- The weight loss was assessed by weighing the samples before and after bio-modification after conditioning at 20 °C for 24 h and at 65% RH.
- The nitrogen content (%N) of the treated and untreated fabric samples was evaluated using the Kjeldahl method^37^.
- The antimicrobial activities of bio-modified post-finished fabrics were evaluated against bacterial strains E. coli ATCC 8379 and Staphylococcus aureus ATCC 25,923 using the colony-forming technique (CFU) according to the reported method by Gupta et al.^38^.
- The UV-protection factor (UPF) of the untreated and treated fabric samples was evaluated according to the Australian/New Zealand Standard Method AS/NZS 4399:1996 and classified as follows: good (UPF: 15–24), very good (UPF: 25–39), and excellent (UPF > 40). The higher the UPF value, the better the UV-protection ability.
- The surface morphology of the selected samples was observed via SEM (Quanta SEM 250 FEG field-emission gun) with an attached energy-dispersive X-ray analysis unit with an accelerating voltage of 30 kV (FEL Co., Netherlands).
- The scent intensity rate (SIR) of bio-modified post-scent-finished samples was evaluated by a well-trained test panel^39^.
-Surface roughness of untreated and treated fabric samples was determined using Surface roughness tester SA6260 (SAMA Tool, Italy).

## Results and discussion

### Molecular identification of the fungal isolate

**Fig. 1:**
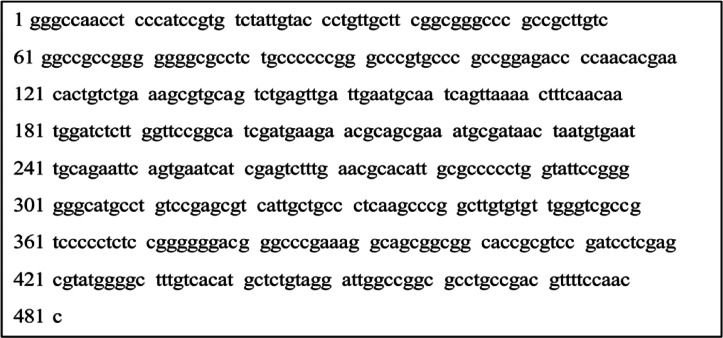
Aligned sequence data (481 bp) of 18 S rRNA amplified from the fungal strain.

**Fig. 2:**
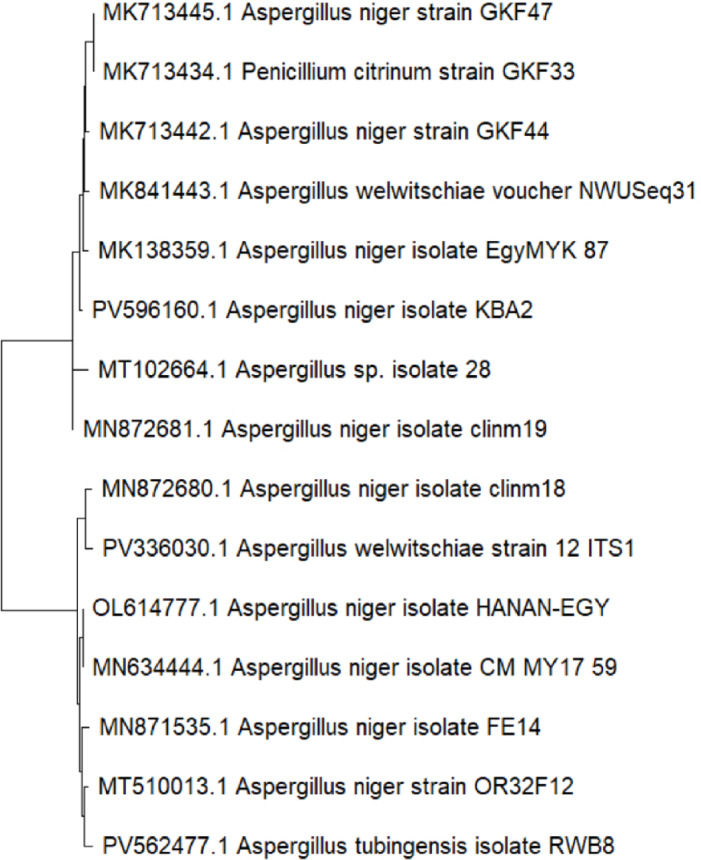
Phylogenetic trees showing the relationship of the fungus *Aspergillus niger* isolate HANAN-EGY with other related fungal species retrieved from GenBank based on their sequence homologies of 18 S rRNA.

The fungal isolate performed DNA amplification for molecular identification using a universal fungal primer set internal transcribed spacer ITS1/ITS4^40^. The PCR products were sequenced, processed, and submitted to GenBank. Figure 1 shows a nucleotide sequence of 481 bp of the fungal sp. isolate’s whole 18 S rRNA gene, which was determined in both strands. BLAST analysis of its specific amplicons showed a 100% identity with *Aspergillus niger* strain OR32F12 (accession number: MT510013.1). Thus, the isolate was identified as a strain of *A. niger* based on both morphological and molecular studies. The isolate rRNA gene sequence was submitted to the GenBank under the accession number OL614777 and designated as *A. niger* HANAN-EGY. The sequence was registered in the GenBank https://www.ncbi.nlm.nih.gov/nuccore/OL614777.The phylogenetic tree of this fungal isolate was also constructed in Fig. 2.

### One-factor-at-a-time optimization

**Fig. 3:**
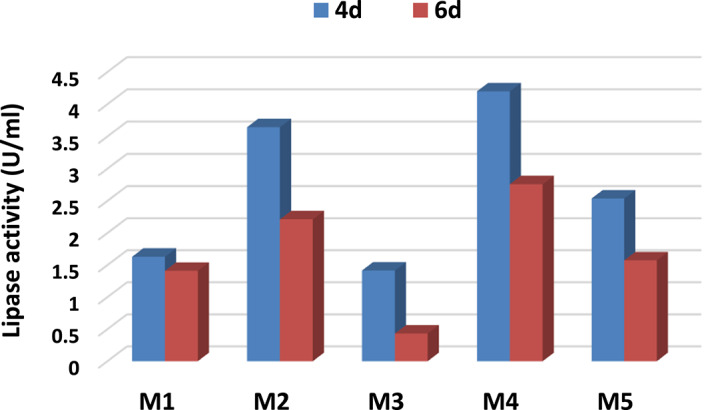
Effect of different media on lipase production by *A. niger* HANAN-EGY.

Figure 3 shows the effect of different media on lipase production by *A. niger* HANAN-EGY at two different time points (4 and 6 days). It indicates variations in lipase activity among the tested media, highlighting that media composition has an impact on lipase production. For example, M4 and M2 exhibit higher lipase activity compared to other media at both time points, suggesting that these media are more favorable for lipase production. Moreover, lipase production tends to decrease from 4 to 6 days when comparing activity values at different time points for each medium. M4 shows the highest lipase activity after 4 days. This may be linked to the elevated nitrogen content present in M4, as highlighted by Riyadi et al., who noted that fungi require relatively higher nitrogen concentrations for optimal lipase production compared to other microbial enzymes^41^.

**Fig. 4:**
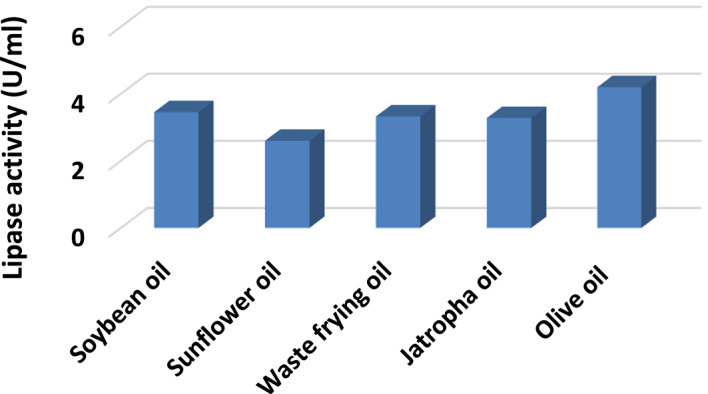
Effect of different oils on lipase production by *A. niger* HANAN-EGY.

Figure 4 shows that the lipase activity values differ among the used oils, indicating that the choice of oil as an inducer has an impact on lipase production by *A. niger* HANAN-EGY. Olive oil exhibits the highest lipase activity (4.2 U/mL), suggesting it is a more effective inducer than the other oils listed. Other oils, such as soybean, waste frying, jatropha, and sunflower oil also show varying lipase activity.

**Fig. 5:**
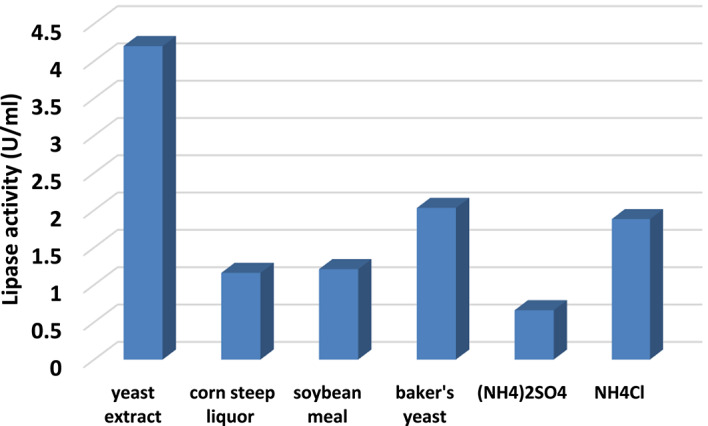
Effect of different nitrogen sources on lipase production by *A. niger* HANAN-EGY.

Figure 5 provides insights into the effect of different nitrogen sources on lipase production by *A. niger* HANAN-EGY. It highlights variations in lipase activity among the different nitrogen sources, suggesting that the choice of nitrogen source plays a crucial role in determining the level of lipase production. The high lipase activity observed for yeast extract indicates its efficacy as a nitrogen source for lipase production in this context. The findings align with the outcomes achieved by Alabdalall et al.^42^, who concluded that yeast extract served as the most effective nitrogen source for lipase production by *A. niger* MH078571.1, *A. niger* MH111398.1, *A. niger* MH111400.1, *A. niger* MH078565.1, and *A. niger* MH079049.1 strains isolated from oil seeds.

### Central composite orthogonal (CCO) design

**Fig. 6:**
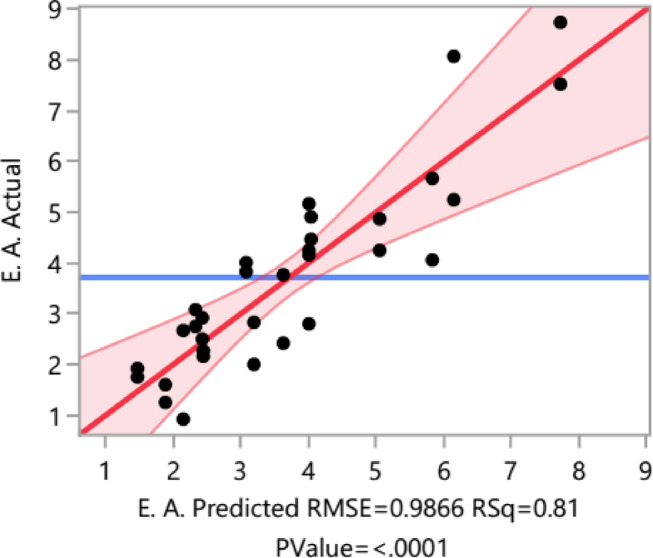
Predicted versus actual.

Using a CCO design offers numerous benefits, including orthogonality, balanced representation, efficient parameter estimation, decreased experimentation time and cost, simplified analysis, and flexibility in factor levels. These advantages render CCO design a valuable option for conducting experimental design and analysis efficiently and effectively^43^. A response surface CCO was created for three continuous factors: WB (X_1_) concentration, YE (X_2_) concentration, and OO (X_3_) concentration. The experimental design for the CCO involved 32 runs, with three variables each having five levels (Table 2). Our experimental findings closely aligned with the predicted values, as shown in Table 2; Fig. 6.

**Table 2:** CCO design and response.

Run	Pattern	X_1_ (WB)	X_2_ (YE)	X_3_ (OO)	E. A. (U/ml)	
		(%)	(%)	(%)	Actual	Predicted
1	a00	3.57	4.5	2	4.06	5.84
2	a00	3.57	4.5	2	5.67	5.84
3	−−−	5	3.5	1	2.16	2.44
4	−−−	5	3.5	1	2.27	2.44
5	−−+	5	3.5	3	7.53	7.74
6	−−+	5	3.5	3	8.75	7.74
7	−+−	5	5.5	1	3.08	2.33
8	−+−	5	5.5	1	2.75	2.33
9	−++	5	5.5	3	8.08	6.16
10	−++	5	5.5	3	5.25	6.16
11	0a0	10	3.21	2	4.91	4.04
12	0a0	10	3.21	2	4.47	4.04
13	00a	10	4.5	0.71	1.92	1.47
14	00a	10	4.5	0.71	1.75	1.47
15	0	10	4.5	2	5.17	4.01
16	0	10	4.5	2	4.16	4.01
17	0	10	4.5	2	2.8	4.01
18	0	10	4.5	2	4.25	4.01
19	00 A	10	4.5	3.29	4.87	5.06
20	00 A	10	4.5	3.29	4.25	5.06
21	0A0	10	5.79	2	2.83	3.19
22	0A0	10	5.79	2	2	3.19
23	+−−	15	3.5	1	1.25	1.88
24	+−−	15	3.5	1	1.6	1.88
25	+−+	15	3.5	3	3.77	3.62
26	+−+	15	3.5	3	2.42	3.62
27	++−	15	5.5	1	2.67	2.15
28	++−	15	5.5	1	0.92	2.15
29	+++	15	5.5	3	2.5	2.43
30	+++	15	5.5	3	2.92	2.43
31	A00	16.44	4.5	2	4.01	3.08
32	A00	16.44	4.5	2	3.83	3.08
Where E. A. is the enzyme activity.						

The ANOVA analysis table provides information on the statistical significance of the factors and interactions in the CCO design, specifically regarding the effect of WB, YE, and OO concentrations on lipase activity (Table 3). The linear effect of WB concentration on lipase activity is significant (X_1_, F Ratio = 26,71, *p* <.0001). Similarly, the linear effect of OO concentration is highly significant (X_3_, F Ratio = 45.15, *p* <.0001). However, the WB, YE, and OO interactions do not show significant effects, except that of WB and OO is significant (X_1_*X_3_, F Ratio = 12.91, *p* =.0016).

**Table 3:** ANOVA analysis results.

Source	Coefficient estimate	Degree of freedom	Sum of Squares	F Ratio	*p*-value
Model	4.008	9	90.889	10.375	< 0.0001*
X_1_	−1.072	1	25.995	26.707	< 0.0001*
X_2_	−0.329	1	2.444	2.511	0.1273
X_3_	1.394	1	43.950	45.153	< 0.0001*
X_1_ × _2_	0.095	1	0.144	0.148	0.7038
X_1_ × _3_	−0.886	1	12.567	12.911	0.0016*
X_2_ × _3_	−0.366	1	2.146	2.205	0.1518
X_1_^2^	0.271	1	0.807	0.829	0.3724
X_2_^2^	−0.236	1	0.611	0.628	0.4367
X_3_^2^	−0.450	1	2.225	26.707	0.1448
Lack Of Fit		5	9.192	2.557	0.0669
Pure Error		17	12.222		
Total Error		22	21.414		
Cor total		31	112.303		
*R*^2^ = 0.81 Adj *R*^2^ = 0.73 Pred *R*^2^ = 0.98 *CV* = 0.26					

In terms of lipase production, any response with a p-value less than 0.05 was deemed significant. The statistical analysis revealed that a high F-ratio of 10.37 indicates that the model explains a significant portion of variability, supported by a very low p-value of less than 0.0001, highlighting the importance of the independent variables. In contrast, the low F-ratio of 2.5 for lack of fit, along with a p-value of 0.07, suggests that the model adequately captures variability without significant unexplained patterns. The analysis confirms the model’s statistical significance and its ability to explain variation in the dependent variable, supported by a strong coefficient of determination (*R*² = 0.81). This value exceeds the 0.75 threshold, indicating model suitability^44^. The R² value of 0.809 is consistent with the adjusted *R*² (adj *R*² = 0.73), confirming a good fit for the quadratic model. The predicted *R*² of 0.98 is also in reasonable agreement with adj *R*², differing by about 0.20, demonstrating that the model effectively captures the relationship between the independent and response variables^45^. The coefficient of variation (CV) serves as a relative indicator of dispersion, thereby aiding in the evaluation of the extent of error or variability present in the model concerning the scale of the data. CV of 26.5% indicates that the standard error constitutes approximately 26.5% of the mean response, reflecting a moderate degree of relative variability. A reduced CV would imply more precise model predictions concerning the mean.

**Fig. 7:**
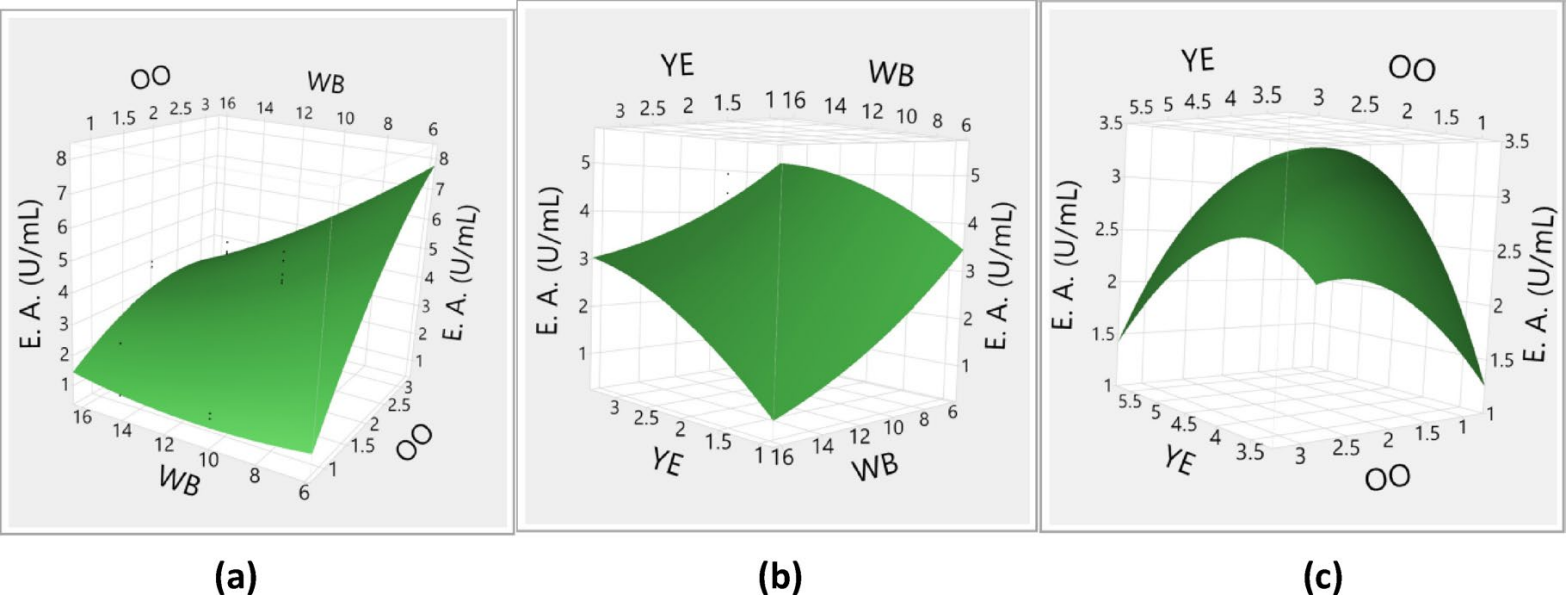
Response surface plots (3D) showing the effects of variables on the response.

Figure 7a illustrates the response surface plots that depict the relationship between OO and WB concentrations concerning lipase activity. It is observed that lipase activity increases as the concentration of OO rises from 1% to 3%. Conversely, an increase in WB concentration from 5% to 15% results in a decline in enzyme activity. Figure 7b presents the response surface plots that explore the interaction between YE and WB concentrations concerning enzyme activity. Additionally, Fig. 7c highlights the impact of varying YE and OO concentrations on lipase activity. The analysis indicates that variations in YE concentrations have a limited impact on lipase activity. In contrast, significant fluctuations in lipase activity are noted with changes in the concentrations of both OO and WB.

Utilizing multiple regression analysis on the experimental data delineates the empirical relationship between the response variable and the independent variables through a second-order polynomial equation [Eq. 1] expressed in terms of coded factors:

1 $${\rm E.A. (U/ml) = 4.008 - 1.072X_1 - 0.329X_2 + 1.552X_3 + 0.095X_1X_2 - 0.886X_1X_3 - 0.366X_2X_3 - 0.271X_1^2-0.235 X_2^2 -0.450 X_3^2}$$

Where E. A. (U/ml) is the enzyme activity; X_1_, X_2_, and X_3_ are the coded values of WB, YE, and OO, respectively. The positive coefficients associated with X_3_ = OO suggest that the linear effect of OO enhances the response, whereas the coefficients for X_1_ = WB and X_2_ = YE reduce the response. Furthermore, all the squared coefficients contribute to a decrease in the response.

The highest yield of lipase was achieved with a fermentation medium consisting of 5% WB, 3.5% YE, and 3% OO. To confirm the accuracy of the model’s predictions, the specified optimal conditions were applied within the regression model [Eq. 1], and the enzyme activity was computed. The predicted enzyme activity (EA) using the given optimal conditions was 7.35 U/mL. This matches very well with the experimental values at similar conditions: Runs 5 & 6 (X1 = 5, X2 = 3.5, X3 = 3) → Observed EA = 7.53 and 8.75. So, the model prediction is accurate and validated within experimental variation. Once the process parameters were optimized on a small scale, lipase production was commenced on a larger scale to maximize enzyme production.

### Pilot-scale production of lipase

**Fig. 8:**
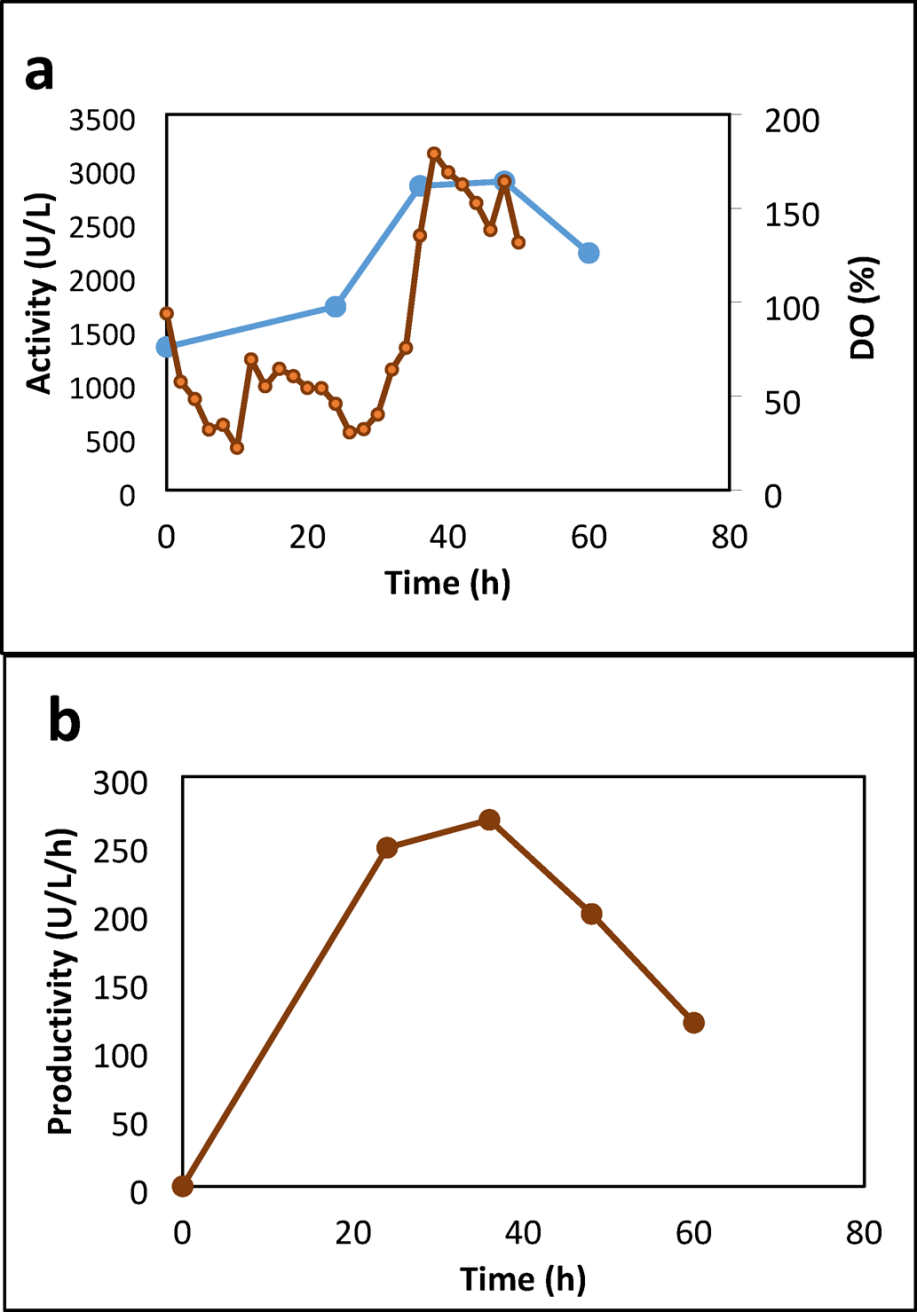
Time courses for lipase activity (a), dissolved oxygen (a), and productivity (b), in a controlled bioreactor operating at 200 rpm, 1 vvm, 30 °C, and initial pH 7.

The BioFlo 310 bioreactor (New Brunswick Scientific) was utilized for the batch culture, with a culture volume of 4 L of an optimized medium of 5% WB, 3.5% YE, and 3% OO. The operating conditions for the bioreactor included a pH of 7, a temperature of 30 °C, agitation at 200 rpm using dual six-blade Rushton impellers with a diameter of 75 mm, and an airflow rate of 4.0 L/min (equivalent to 1 volume of air per volume of culture per min). Throughout the experiment, samples were taken at regular intervals and subjected to analysis for parameters such as total dry mass, reducing sugar content, total protein concentration, and lipase activity. As shown in Fig. 8, the lipase activity in the bioreactor increases over time, reaching a maximum at 48 h with a value of 2875 U/L, then slightly decreases to 2208.333 U/L at 60 h. The productivity of lipase also follows a similar trend to the lipase activity. However, it reaches its maximum value of 268.3245 U/h at 36 h and then decreases sharply to 199.4172 U/h at 48 h.

In general, the findings indicate that the levels of dissolved oxygen affect the production of lipase in the controlled bioreactor. The enhanced levels of DO observed at 48 h align with the peak lipase activity. This association implies that ensuring a sufficient oxygen supply during fermentation is essential for optimal lipase production.

In the next experiment, we used the DO cascade system to facilitate the automatic introduction of oxygen into the medium. Simultaneously, the agitation is connected in a cascade manner to the DO, allowing for the adjustment of agitation speed between the minimum (200 rpm) and the maximum set point of agitation (600 rpm). This ensures that the set percentage of DO remains at or above 30%. Similarly, the aeration is cascade-connected to the DO, varying between 1 and 2 vvm (4–8 L/M) to maintain the DO percentage at no less than 30%. 4 L of a modified culture medium of 5% WB, 3.5% YE, and 2% OO is used. The OO concentration in the current experiment is reduced due to the excess oil left over from the prior experiment.

**Fig. 9:**
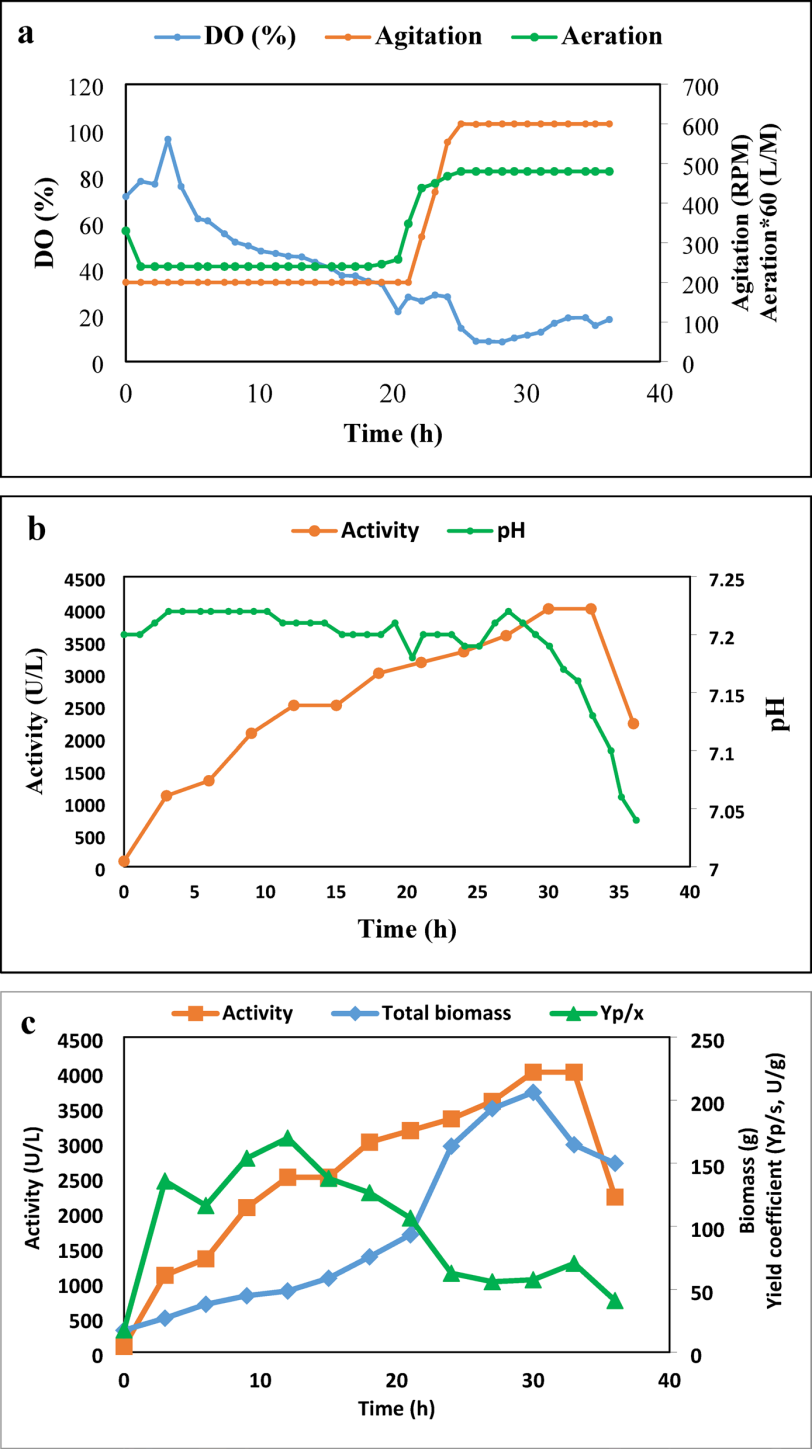

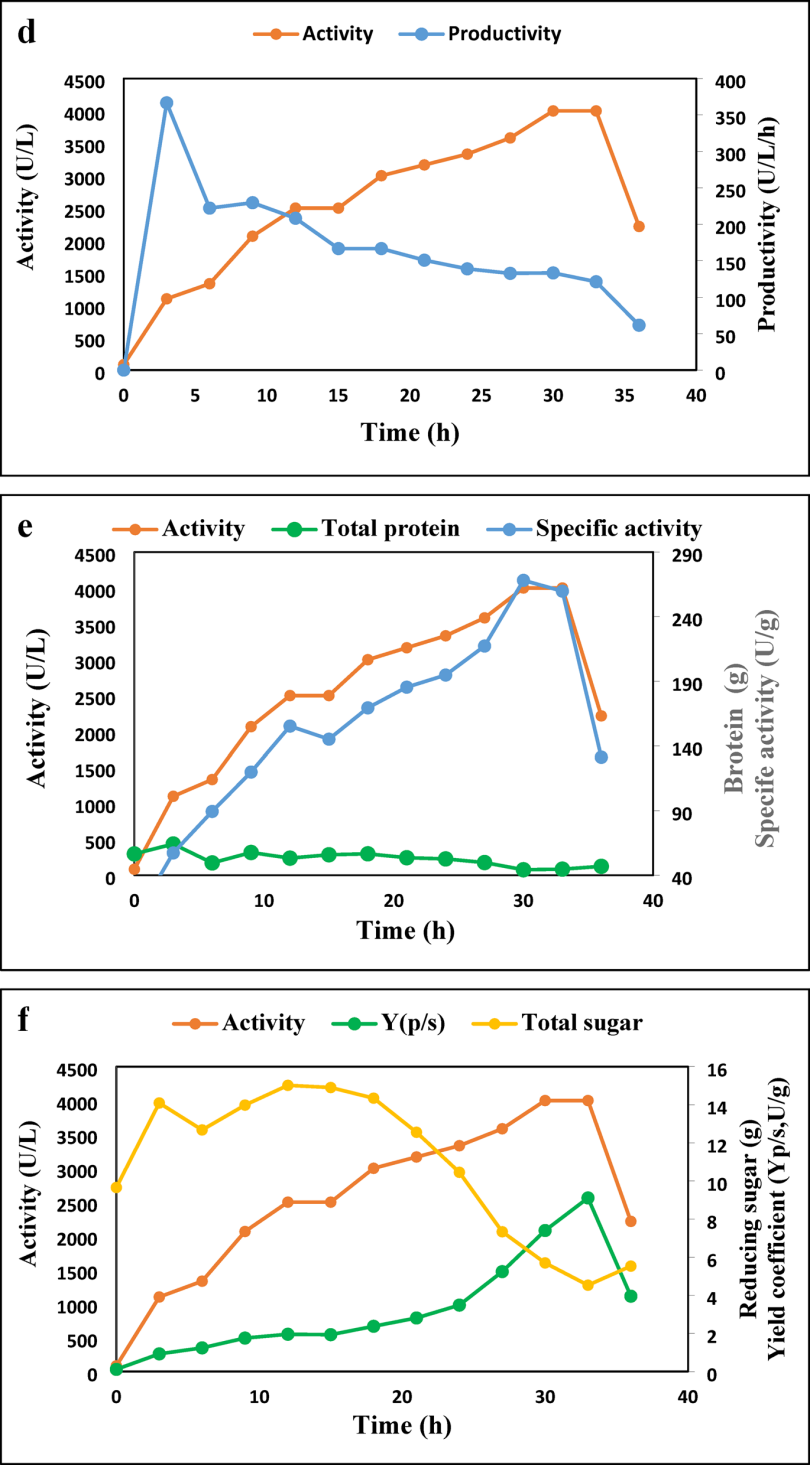
Kinetics of DO, agitation, and aeration (**a**), lipase activity and pH (**b**), total biomass and yield coefficient (**c**), productivity (**d**), total protein and specific activity (**e**), and total sugar and yield/g sugar (**f**) of the *A. niger* HANAN-EGY in the culture with DO cascade system.

Table 4 presents the time course of various fermentation parameters related to the lipase activity of the *A. niger* HANAN-EGY strain in a controlled bioreactor with a DO cascade system. Changes in DO, agitation, and aeration are presented in Fig. 9a. The levels of DO fluctuate during the fermentation process. Initially, it begins at 71.4% and rises to a maximum of 96.2% after 3 h. Subsequently, the DO levels steadily decline, dropping to 8.7% after 27 h. At this stage, the agitation and aeration have both reached their maximum levels of 600 rpm and 8 L/M, respectively. These variations in DO levels suggest shifts in oxygen availability during fermentation, which may influence the growth and the lipase activity of the *A. niger* HANAN-EGY strain. The initial pH is 7.2, and a relatively stable pH is observed throughout the fermentation process. However, at 27 h, the pH experienced a slight drop from 7.22 to 7.04 by 36 h (Fig. 9b). The lipase activity increases over time, starting from 83.33 U/L at 0 h and reaching a maximum of 4000 U/L at 30 h. This level of activity is attained when both agitation and aeration are at their maximum levels of 600 rpm and 2vvm, respectively. The escalation in agitation speed and aeration improves oxygen transfer and mixing in the bioreactor, consequently boosting lipase activity to 4000 U/L in comparison to the previous trial (activity 2875 U/L) conducted at 200 rpm and 1 vvm. The findings support the conclusion that higher dissolved oxygen concentration in the medium increases lipase activity^46^. Furthermore, the fermentation period has been shortened from 48 h to 30 h, and the fermentation medium has become more cost-effective with the olive oil concentration lowered to 2% from 3%. Thereafter, the lipase activity decreases to 2216.67 U/L at 36 h.

There has been a notable reduction in the dissolved oxygen levels in the medium, which has consistently shown a positive correlation with the growth of biomass (Fig. 9C), and frequently with an elevation in enzyme activity (Fig. 9d). The medium started with high oxygen levels, but as the microorganism population grew, it decreased rapidly from 6 to 21 h. At 33 h, oxygen levels increased as the microorganism’s growth phase ended and oxygen consumption decreased. Consequently, DO is a critical factor in monitoring fermentation. It initially decreased rapidly, reaching levels as low as 8–11% saturation and remaining constant for about 3 h. Then, there was an increase in DO levels, indicating the end of cell growth and the onset of lipase depletion, making it a more reliable indicator of fermentation progress, especially in the context of lipase production^47^.

**Table 4:** Time course of different fermentation parameters concerning lipase activity of the *A. niger* HANAN-EGY in a controlled bioreactor with DO cascade system.

Time	DO	Agitation	Aeration	*p* H	Lipase Activity	VP	Total biomass	Y(*p*/x)	RX	Total protein	Specific activity	Total sugar	Y(*P*/S)
(h)	(%)	rpm	L/M		U/L	(U/L/h)	(g)	(U/g	(g/h)	(g)	(U/g)	g	(U/g)
0	71.4	200	4	7.20	83.33	0	17.45	17.66	0	56.49	5.45	9.65	31.91
3	96.2	200	4	7.22	1100.00	1121.83	27.03	135.87	3.19	64.15	57.26	14.09	260.68
6	60.9	200	4	7.22	1333.33	686.25	38.03	116.37	3.42	49.50	89.39	12.67	349.07
9	50.1	200	4	7.22	2066.67	732.77	44.84	153.94	3.04	57.59	119.86	13.98	493.70
12	45.6	200	4	7.21	2500	662.75	48.61	169.94	2.59	53.21	155.24	15.01	550.04
15	40.3	200	4	7.2	2500	519.64	58.75	137.92	2.75	55.83	145.11	14.90	543.64
18	34.8	200	4	7.2	3000	515.11	75.69	126.57	3.23	56.56	169.38	14.34	667.82
21	27.9	315	5.8	7.2	3166.67	458.00	93.28	106.40	3.61	53.54	185.38	12.55	790.52
24	28	545	7.8	7.19	3333.33	414.28	163.53	62.68	6.08	52.62	194.79	10.45	980.49
27	8.7	600	8	7.22	3583.33	389.26	193.32	55.96	6.51	49.79	217.24	7.33	1474.84
30	11.4	600	8	7.19	4000	385.05	206.19	57.51	6.29	44.23	268.14	5.69	2081.15
33	18.9	600	8	7.13	4000	342.37	164.57	70.52	4.45	44.68	259.74	4.53	2561.92
36	18.2	600	8	7.04	2216.66	162.28	149.90	41.02	3.67	46.86	131.24	5.53	1111.75

The identified culture parameters include volumetric productivity (VP), product-biomass yield (Y(P/X)), rate of biomass yield (RX), specific enzyme activity, and product-substrate yield (Y(P/S) (Table 3). The highest Y(P/X) coefficient was observed at 12 h, rpm 200, and 1vvm, reaching 169.94 U/g, accompanied by a VP of 2066 U/L. The highest biomass of 206 was reached after 30 h, coinciding with the maximum VP (4000 U/L). Similarly, the highest specific enzyme activity of 296 U/g protein was obtained after 30 h (Fig. 9e). The maximum RX (6.51 g/h) is reached at 27 h, accompanied by 3584.33 U/L VP. Total sugar indicates the amount of sugar present in the fermentation medium. As shown in Fig. 9f, as the reduced sugar consumed over time reached its minimum value of 4 g at 33 h, Y(P/S) increased gradually, reaching its peak at the same time.

### Bio-modification using the locally produced fungal lipase enzyme

For a given set of enzymatic treatments of polyester (100%), cotton/polyester (50/50), and wool/polyester (50/50) fabrics using the locally produced lipase enzyme, the data in Table 5 revealed that:

Bio-modification of polyester, cotton/PET (50/50), and wool/polyester (50/50) fabrics results in a loss in weight, %N, especially PET/wool blend, along with an improvement in the smoothness of the bio-modified substrates, expressed as a decrease in Ra values.

 The change in the aforementioned properties reflects the catalytic effect of lipase on the hydrolysis of ester bonds along PET macromolecules [Eq. 2]^25,48^, removing lipid barriers from the pretreated wool fiber surface^49^, as well as removing remnant fatty matter from the pretreated cotton component^50^.


2


The extent of variation in the abovementioned properties is governed by fabric type, weight, structure, surface morphology, amorphous to crystalline ratio, lipase-substrate complex, mode of interaction, and extent of interaction/enzymatic attack^51,52^.

**Table 5:** Effect of bio-treatment using biosynthesized lipase enzyme on some performance properties of treated fabric samples.

Fabric type	Treatment condition	WL (%)	%*N*	Ra (µm)
Polyester (100%)	untreated	--	--	15.40
	With enzyme	0.199	--	13.43
Cotton/PET (50/50)	untreated	--	--	16.84
	With enzyme	0.736	--	14.06
Wool/PET (50/50)	untreated	--	7.488	23.41
	With enzyme	2.33	7.092	16.16

Enzymatic treatment condition: lipase enzyme conc. (75 mL enzyme/150 H_2_O); MLR 1/20 at 50 °C for 60 min using IR-dyeing machine.

### Sustainable value-added post-functionalization of bio-modified substrates

 As far as the change in add-on (%), nitrogen content (%N) surface roughness (Ra), scent intensity rate (SIR), UV-protection factor (UPF), and antibacterial activity of bio-modified post-functional finished polyester-containing fabrics, the data in Table 6 signify that post-treatment of lipase bio-modified substrates with any of the used functional additives is accompanied by a decrease in %N, especially of the treated polyester/wool fabric, a slight increase in surface roughness, a noticeable improvement in scent intensity of vanillin-loaded substrates, an increase in UV-protection efficacy along with a significant improve in antibacterial functionality. The imparted antibacterial activity against *E. coli* and *S. aureus* pathogenic bacteria of the vanillin post-finished substrates via their active sites and/or ester-crosslinking is attributed to its ability, as a phenolic aldehyde, to disrupt and damage the pathogenic bacterial cells, thereby adversely affecting their growth and survival. On the other hand, the enhanced UV-protection functionality, expressed as UPF value, reflects the ability of vanillin-loaded substrates to adsorb the harmful UV-radiation^26,39,53–55^. On the other hand, fixation of ZnONPs onto/within the lipase-modified substrates via their active sites and/or ester-crosslinking using CA/SHP, and their photocatalytic activity and generation of active species, ROS, like ^•^OH, ^•^O_2_^-^, H_2_O_2_, etc., liberation of Zn^2+^ ions along with ZnONPs mechanical action leading to the death of pathogenic bacteria [Eqs 3–6]^20,21,56,57^. Additionally, the substantial improvement in the imparted UV-protection reflects the capability of the immobilized and fixed ZnONPs in absorbing, scattering, blocking, and shielding the harmful UV-B radiation^6,20,58^. The generation of the ROS Tentative mechanism as follows:

3 $$\:ZnONPs-loaded\:substrate+{h}_{\nu\:}\:\underrightarrow{{H}_{2}{O}^{}}\:ZnO{(h}^{+})+\:ZnO{(e}^{-})$$

where h^+^ and e^-^ are a positive hole and an electron, respectively

4 $$\:{h}^{+}\:+\:\:{H}_{2}O\:\:\to\:{}^{\bullet\:}OH+\:{H}^{+}$$

5 $$\:{e}^{-}+\:{O}_{2}\to\:\:\:{{}^{\bullet\:}O}_{2}^{-}$$

6 $$\:{{}^{\bullet\:}O}_{2}^{-}+\:{H}^{+}\:\to\:\:{HO}_{2}^{\bullet\:}$$

$$\:2H{O}_{2}^{\bullet\:}\:\to\:\:{H}_{2}{O}_{2}+2\left[O\right]$$

The change in the imparted functional properties mentioned above is governed by the type of functional additive and its extent of fixation and immobilization onto/within the bio-modified substrates, its antibacterial activity and anti-UV-functionality as well as kind of substrate, the extent of its bio-modification, availability, and accessibility of its reactive sites, e.g. ‒OH, ‒COOH, or ‒NH_2_, etc., as well as the mode of interaction and extent of fixation during the microwave fixation step^8,48^. Immobilization and fixation of the functional additives, namely ZnOMPs and vanillin onyo and/or within the ester crosslinked fabric structure via:

i) Electrostatic interaction among ‒OH‾, ‒COO‾ of PET and ester crosslinked cotton and $\:-{}^{+}N{H}_{2}$ of wool structure under acidic pH,
ii) Hydrogen bonds among ‒COOH and ‒OH of ester crosslinked substrates and functional groups of the used additives,
iii) chemical bonds among vanillin functional groups, e.g. ‒CHO, ‒OH and OCH_3_, and the wool component active sites, e.g. ‒NH_2_, ‒OH, ‒COOH,
iv) Coordination bonds among the functional groups such as ‒COOH, ‒OH, ‒NH_2_ groups and the ZnONPs.

The experimental results also demonstrate that a combination of vanillin and ZnONPs functional additives has an excellent synergetic effect and significantly improves the imparted UV-protection functionality and anti-bacterial property against the pathogenic G + ve and G-ve bacteria, regardless of the treated substrate.

**Table 6:** Effect of enzymatic treatment using lipase enzyme followed by post-finishing with Vanillin and/or ZnONPs on some performance and functional properties of the finished polyester containing fabric samples.

Fabric type	Post –finished Conditions	Add on (%)	%N	Ra (µm)	SIR	UPF	Anti-bacterial activity (%)	
							G + ve	G-ve
**Polyester (100%)**	Vanillin	8.015	--	14.62	+++	43	67.66	51.26
	ZnONPs	4.335	--	17.11	--	51	55.38	49.27
	Vanillin + ZnONPs	4.620	--	15.19	++	74	73.16	51.42
	Blank	---	--	13.43	--	30	--	--
**Cotton/PET** **(50/50)**	Vanillin	5.567	--	14.85	+++	138	67.66	56.64
	ZnONPs	2.936	--	16.45	--	114	61.93	55.13
	Vanillin + ZnONPs	5.124	--	16.39	++	160	73.53	57.90
	Blank	---	--	14.06	--	28	--	--
**Wool/PET** **(50/50)**	Vanillin	9.307	6.563	15.58	+++	32	69.58	60.07
	ZnONPs	3.931	6.198	17.12	--	25	58.73	49.16
	Vanillin + ZnONPs	8.212	6.443	16.50	++	49	74.34	64.07
	Blank	---	7.092	16.16	--	13	--	--

Enzyme dose (75 mL enzyme/150 mL H_2_O_2_).

Post-finishing condition: vanillin (10 g/L), ZnONPs (10 g/L), citric acid (20 g/L), SHP (10 g/L), MLR (1/20), at 30 °C, 20 min, using ultrasonic bath followed by microwave fixation at 400 W/6min.

WL: weight loss, Ra: surface roughness, SIR: Scent intensity, UPF: UV-Protection Factor.

The data in Table 6 also demonstrate that the change in the aforementioned physicochemical, mechanical and functional properties as a function of additive type, keeping the textile substrate fixed, follows the decreasing order:.

- Add on: vanillin > vanillin/ZnONPs > ZnONPs.

- %N: None > vanillin > vanillin/ZnONPs > ZnONPs.

- Ra: ZnONPs > vanillin/ZnONPs > vanillin > None.

- SIR: vanillin > vanillin/ZnONPs.

- UPF: vanillin/ZnONPs > vanillin > ZnONPs > None.

- Antibacterial activity: vanillin/ZnONPs > vanillin > ZnONPs > None, keeping the functional additive fixed,.

The data in Table 6 also signify that the imparted antibacterial activity against the tested bacteria follows the decreasing order *S. aureus* > *E. coli*, reflecting their differences in cell constitution, physiology and metabolism^59^. As shown in Table 6 the inclusion of vanillin as an eco-friendly functional additive in post-finishing formulation exhibited a pleasant aroma, expressed as SIR value, regardless of the substrate used. Furthermore, the experimental results, Table 6, demonstrate that incorporation of ZnONPs into the finishing formulation, alone and in combination with vanillin, is accompanied by a slight increase in surface roughness of the post-finished substrates. The extent of variation in the abovementioned performance and functional properties is governed by fabric type as well as post-finishing constituents, as mentioned before.

### SEM and EDX analysis

**Fig. 10:**
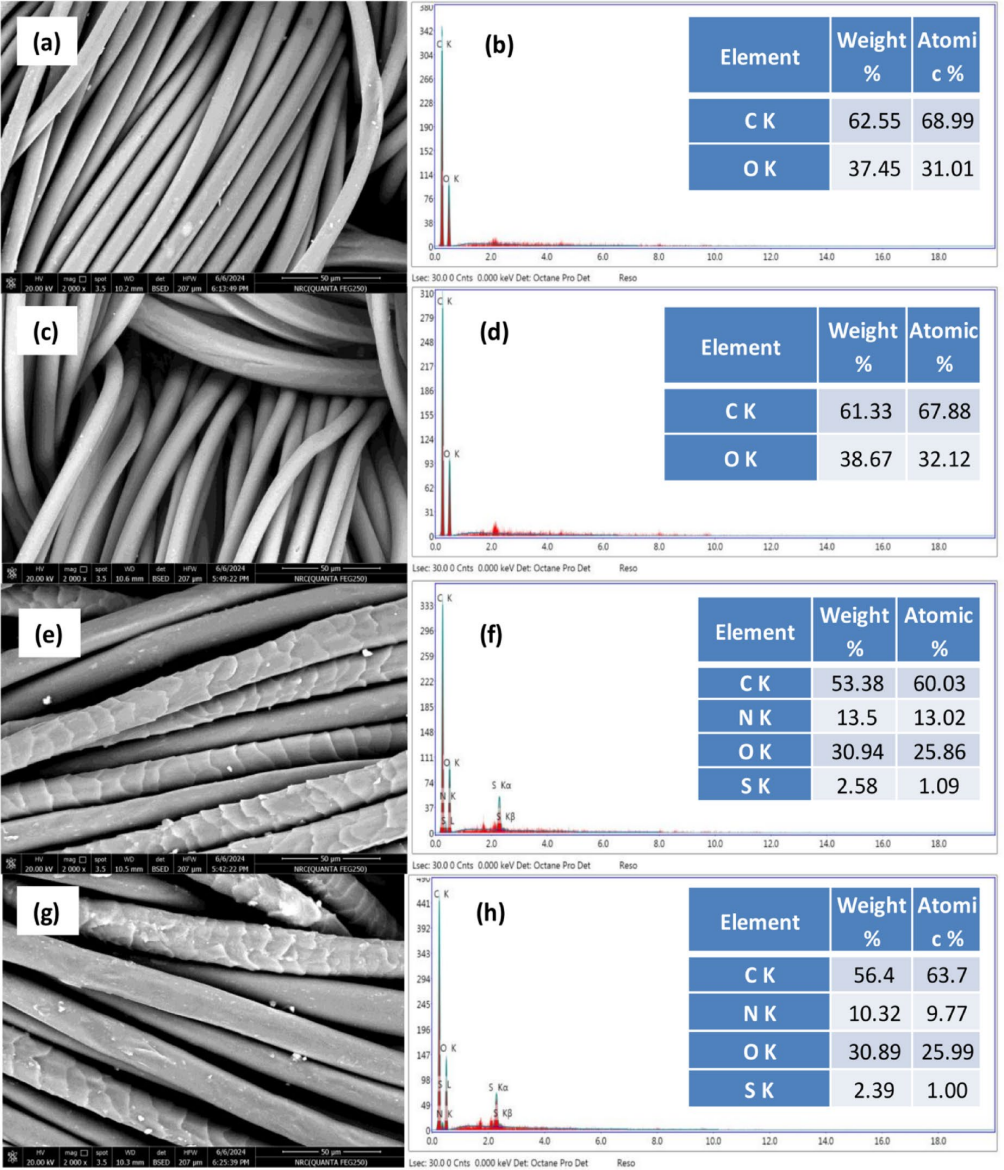
SEM and EDX spectra of untreated polyester (100%) (a&b), lipase-treated polyester (c&d), untreated wool/polyester (50/50) (e&f), lipase-treated wool/polyester (50/50) (g&h).

The surface morphology (SEM) and EDX spectra of untreated 100% polyester and lipase bio-modified samples are presented in Figs. 10 (a&b) and (c&d) respectively. Figure 10c, showed few pits and voids compared to that of the untreated PET fabric, Fig. 10a. However, the surface smoothness of PET fabric did not change significantly, despite the voids and cracks, because the weight loss of PET fabrics after lipase treatment was marginal (0.199%). Additionally, these voids and pits on the PET fabrics could enhance the wettability of the bio-modified fabric samples^60^.

Moreover, Fig. 10 (e&g) represents the SEM of untreated wool/PET and lipase-treated wool/PET samples, respectively. Partial removal of wool scales compared with the untreated sample was observed. The EDX spectra and the elemental analysis Fig. 10h showed a decrease in Sulphur content for the lipase-treated wool/PET as a direct consequence of the partial removal of wool scales, compared with the untreated one (Fig. 10f).

**Fig. 11:**
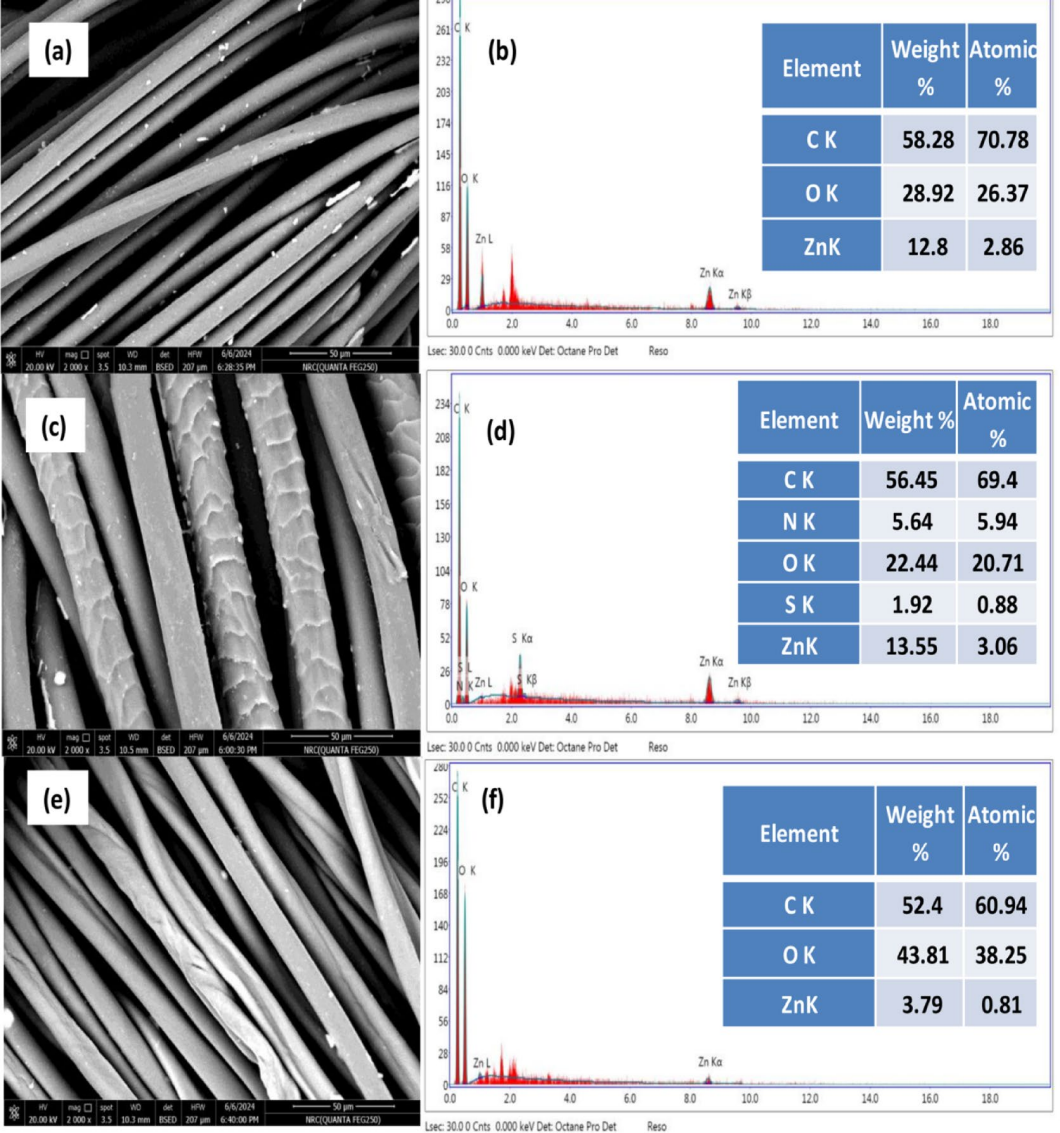
SEM and EDX spectra of enzymatic treatment with bio-prepared lipase followed by post-finishing with ZnONPs of polyester (100%) (a&b), wool/PET (50/50) (c&d), and Cotton/PET (50/50) (e&f).

The SEM and EDX spectra of PET (100%), wool/PET (50/50) and cotton/PET (50/50) fabric samples that were bio-modified with lipase and subsequently nano-finished with ZnONPs are shown in Fig. 11 (a&b), (c&d), and (e&f), respectively. Both the SEM images and EDX spectra confirm the deposition of Zn element onto the fabric sample surface; the extent of deposition is governed by the type of substrate, where the wool/PET samples showed higher content of Zn element.

## Conclusion

- The locally isolated *Aspergillus niger* HANAN-EGY strain was employed for lipase production utilizing a cost-efficient approach. Following initial small-scale optimization, the production process was scaled up in a BioFlo 310 bioreactor, resulting in a reduction of both fermentation medium concentration and duration to 30 h, while achieving an increased lipase yield of 4000 U/L.
- Enzymatic treatment using locally produced lipase as an eco-friendly bio-catalyst was carried out to modify the fabric surface like wool, polyester, wool/polyester, and polyester/cotton fabrics as well as to create active sites on their surface at mild conditions, which in turn can form bonds with the used functional additives, namely vanillin and/or ZnONPs in post-treatment for imparting multifunctional properties to the bio-modified post-finished substrate, like antibacterial, anti-UV, stain-release, and pleasant smell.
- The suggested and implemented sustainable bio-modified post-functional finishing and pollution abatement strategy, taking into consideration product quality, functionality, and economic and ecological concerns can be considered as an eco-friendly cleaner production process using environmentally sound emerging bio- and nano-technologies for upgrading textile products quality, high value-added, enhancing their market share locally and globally as well as satisfying the ever-growing consumer demands and ever-increasing environmental concerns.
- The locally produced hydrolytic enzyme as well as its implementation in textile surface pre-modification followed by post-treatment with environmentally sound functional additives, vanillin and/or ZnONPs, can be easily adopted and practiced on a large scale in textile sector, taking in consideration the increasing concerns about environment, sustainability and the ever-growing textile consumer demands for vast potential applications.

## Data Availability

The datasets generated and/or analysed during the current study are available in the NCBI repository, [https://www.ncbi.nlm.nih.gov/nuccore/OL614777.1/](https:/www.ncbi.nlm.nih.gov/nuccore/OP709283.1) with accession number OL614777.1.
